# Stressed telomeres without POT1 enhance tumorigenesis

**DOI:** 10.18632/oncotarget.10600

**Published:** 2016-07-13

**Authors:** Agnel Sfeir, Eros Lazzerini Denchi

**Affiliations:** Department of Molecular and Experimental Medicine, The Scripps Research Institute, La Jolla, CA, USA

**Keywords:** telomere, genomic stability, POT1, replication stress, ATR, Autophagy

Chromosome ends in mammalian cells are protected by a specialized 6 subunit complex called shelterin. Mutations in one component of this complex, POT1, have been associated with a variety of cancers including solid and lymphoid tumors [1]. Interestingly, the vast majority of the POT1 mutations cluster within its DNA- binding domains, the so-called OB-fold domains. We have recently uncovered the molecular mechanism by which these mutations contribute to cancer progression [2]. Surprisingly we found that not all the cancer-associated POT1 mutations alter the ability of POT1 to bind telomeric DNA. Instead, our study revealed that POT1 mutations compromise telomere replication, leading to telomere dysfunction. Indeed, the expression of these mutations in various cellular systems resulted in frequent stalling of the replication machinery at telomeric repeats, triggering the activation of the ATR kinase, and ultimately leading to increased genome instability [2]. We determined that this is caused by alterations in the recruitment of the CST (CTC1-TEN1-STN1) complex at telomeres. CST is an RPA like complex that acts as a pol-α primase accessory factor [3] that enables replication fork restart at telomeric DNA.

To address the physiological implication of our findings we generated a mouse model in which POT1 was inactivated in the common progenitor lymphoid (CLPs) cells. We chose this compartment because of the predominance of POT1 mutations in cancer of lymphoid origin. Analysis of these mice revealed that, when combined with p53 inactivation, POT1 inhibition resulted in highly aggressive thymic lymphomas. Tumor cells derived from these mice showed increased levels of telomere dysfunction with many end-to-end chromosomal fusions and signs of replication defects at telomeres [2].

The existence of telomere dysfunction in POT1- depleted tumors created a paradox. How do POT1 mutated cells survive despite the high level of telomere dysfunction and the resultant DNA damage signaling? To get insight into this problem, we interrogated the cancer genomes for clues. Specifically, we compared by NGS the transcription profile of tumors derived from POT1-proficient and POT1-deficient tumors. This analysis revealed that the ATR pathway is significantly downregulated in tumors with POT1 mutations. This observation was confirmed in tissue culture based experiments as well as *in vivo*. The impairment of the ATR-pathway in POT1-mutated tumors at first seems counterintuitive, given that increased ATR signaling has been suggested to allow tumor cells to cope with oncogene-induced replication stress [4]. However, it is possible that the attenuation of the ATR signaling pathway enables tumor cells with replication defects to evade checkpoint activation and cell cycle arrest. In agreement with this, reduced ATR signaling in mice is sufficient to increase the incidence of tumors [5]. In addition, somatic mutations affecting the ATR- signaling pathway have been found in human tumors with microsatellite instability [6].

Several questions remain to be addressed to understand the link between replication stress at telomeres and cancer development. First of all, we still do not understand how telomere replication defects and fragility leads to chromosome fusions. Additionally, from a mechanistic point of view, how POT1 mutations impact telomere replication remains unknown. Our data implicate the CST complex in this process. However, additional biochemical and structural analysis are required to elucidate how mutations in POT1 compromise CST function. Next, it will be of interest to define whether other sources of replication stress, particularly other genetic alterations known to affect telomere replication, promote a similar telomere dysfunction-induced genomic instability and increased tumor formation. Finally, and most importantly, it will be crucial to decipher the mechanism(s) by which cancer cells harboring POT1 mutations bypass telomere-driven crisis. Our data suggest that tumors derived from POT1 deficient mice display attenuated ATR signaling and have a high degree of genome instability. But how is ATR signaling attenuated? The Costanzo lab recently showed that supercoiling of repetitive centromeric DNA by Topoisomerase I precludes loading of RPA, and suppresses ATR signaling [7]. It is tempting to speculate that this physiological mechanism might be hijacked by cancer cells experiencing telomeric replication stress to proliferate despite the ongoing replication stress. Future work will define in better details the molecules involved in the ATR attenuation which might be crucial for tumor progression.

**Figure 1:**
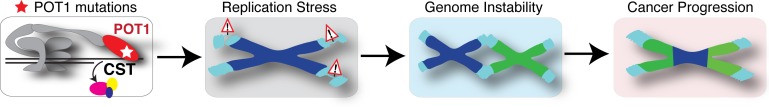
Model depicting the progression from POT1 mutations to aggressive cancer Mutations in the shelterin subunit POT1 impair the function of the CST complex at telomeres, leading to replication-dependent telomere defects (telomere fragility, ATR- dependent signaling, telomere elongation). Altered telomere replication also triggers chromosomal end-to-end fusion that are then subject to breakage-fusion-bridge cycles leading to increased genome instability and more aggressive tumors.
